# Ultrasounds outperform magnetic resonance imaging in quantifying meniscal extrusion in patients with knee osteoarthritis

**DOI:** 10.1002/jeo2.70031

**Published:** 2024-09-30

**Authors:** Fabio Tortorella, Angelo Boffa, Luca Andriolo, Giancarlo Facchini, Maddalena Di Carlo, Marco Miceli, Burt Klos, Stefano Zaffagnini, Giuseppe Filardo

**Affiliations:** ^1^ Applied and Translational Research (ATR) Center, IRCCS Istituto Ortopedico Rizzoli Bologna Italy; ^2^ Clinica Ortopedica e Traumatologica 2, IRCCS Istituto Ortopedico Rizzoli Bologna Italy; ^3^ Radiologia diagnostica ed interventistica, IRCCS Istituto Ortopedico Rizzoli Bologna Italy; ^4^ ICONE Orthopedics and Sports Traumatology Schijndel The Netherlands; ^5^ Faculty of Biomedical Sciences Università della Svizzera Italiana Lugano Switzerland

**Keywords:** extrusion, magnetic resonance, meniscus, osteoarthritis, ultrasound

## Abstract

**Purpose:**

The aim of this study was to quantify meniscal extrusion through ultrasound (US) evaluation in supine and standing positions and to compare the results with those documented through magnetic resonance (MR) imaging in patients affected by knee osteoarthritis (OA).

**Methods:**

Sixty patients (38 men, 22 women, mean age 60.8 ± 9.7 years) with knee OA were enrolled and underwent a 1.5 T MR evaluation and an US examination of the symptomatic OA knee for the evaluation of the medial and lateral meniscus extrusion both in the supine clinostatic position (clino‐US) with the knee fully extended and in the standing weight‐bearing orthostatic position (ortho‐US). For the three imaging evaluations (MR, clino‐US and ortho‐US), both semi‐quantitative and quantitative measurements were performed.

**Results:**

The quantitative analysis documented higher values of medial meniscal extrusion at the ortho‐US evaluation (5.2 ± 2.3 mm) compared to MR (4.2 ± 2.2, *p* < 0.0005) and clino‐US (4.5 ± 2.3, *p* < 0.0005) and of the lateral meniscus at the ortho‐US evaluation (4.3 ± 1.8) compared to MR (3.3 ± 1.6, *p* < 0.0005) and clino‐US (3.8 ± 1.6, *p* < 0.0005). The semi‐quantitative analysis confirmed the same trend for both menisci. Higher extrusion values were documented in women and more advanced OA, as well as in older patients with higher body mass index, the latter being underestimated the most by the MR approach.

**Conclusion:**

US outperforms MR imaging in quantifying meniscal extrusion in patients with knee OA. Moreover, the highest values of meniscal extrusion have been documented using US in standing position compared to the supine position, underlining the importance of the weight‐bearing assessment of meniscal extrusion in knee OA patients.

**Level of Evidence:**

II.

**Abbreviations**
BMI: body mass index
Clino‐US: clinostatic position ultrasound
MR: magnetic resonance
OA: osteoarthritis
Ortho‐US: orthostatic position ultrasound
ROM: range of motion
TKA: total knee arthroplasty

## INTRODUCTION

Knee osteoarthritis (OA) is a common orthopaedic joint disease characterized by macroscopic features such as articular cartilage degeneration, subchondral bone alterations, osteophytes formation, local synovial inflammation and meniscal abnormalities [19]. Among these, in recent years scientific literature focused his attention on meniscal extrusion, a phenomenon which can trigger or exacerbate degeneration processes [21]. Meniscal extrusion is probably a consequence of the complex interactions between joint tissue degeneration and mechanical stresses involved in OA disease [6, 25]. It may cause mechanical joint alterations with a decreased contact area between tibia and femur inducing pathologic loads to the articular surface [2, 14, 15]. This can lead to cartilage and subchondral bone damage, thus contributing to the onset and progression of knee OA [5, 9, 10].

An early diagnosis and treatment of meniscal extrusion could decelerate the evolution of the OA process, possibly postponing further degeneration and reducing the need for total knee arthroplasty (TKA) procedures and the consequent economic burden on the healthcare system [7, 23]. Magnetic resonance (MR) is currently considered the most important imaging modality for identifying meniscal pathologic abnormalities including meniscal extrusion [8], even though it presents the limitation of assessing patients in supine position. In the last years, the use of ultrasonography (US) has expanded as a suitable method for the evaluation of menisci with patients in both clinostatic and orthostatic positions [24]. However, evidence on the potential of using US in patients affected by knee OA for the assessment of the meniscal extrusion, either in a supine or in a standing position, is still limited with respect to the more classic MR approach. The use of US to evaluate meniscal extrusion could provide useful information on this aspect of knee OA, aiding in its diagnosis and potentially facilitating an early treatment and better treatment indications.

The primary aim of this study was to quantify meniscal extrusion through US evaluation in supine and standing positions and to compare the results with those documented through MR imaging in patients affected by symptomatic knee OA. The secondary aim of this study is to investigate any correlation between medial and lateral meniscal extrusion and patients' characteristics.

## MATERIALS AND METHODS

### Study design and patient selection

This study was approved by the Ethics Committee of the IRCCS Istituto Ortopedico Rizzoli, Italy (Prot. No. 0001673), and was registered at ClinicalTrials.gov (NCT06113536). Patients were prospectively enrolled by orthopaedic physicians between January 2021 and January 2023 in a research outpatient clinic specialized in patients with knee OA. Informed consent was obtained from each patient for study participation. Inclusion criteria were male or female patients aged between 18 and 80 years with signs and symptoms of knee OA and radiographic signs of knee OA (Kellgren–Lawrence Grades 1–4). Exclusion criteria were patients unable to express consent, patients who performed knee surgery in the previous 12 months, patients suffering from malignant tumours or rheumatic diseases, patients with a history of a sub‐total or total meniscectomy of the affected knee and patients with axial malalignment >5°. Sixty consecutive patients with knee OA were enrolled according to the inclusion and exclusion criteria. Patients' detailed characteristics are reported in Table 1.

**Table 1:** Baseline characteristics of the included patients.

Gender (male/female)	38/22
Age, y (mean ± SD)	60.8 ± 9.7
BMI, kg/cm^2^ (mean ± SD)	25.8 ± 3.6
Side (left/right)	26/34
Symptoms duration, m (range)	83.3 (6–240)
Kellgren–Lawrence grade	Grade 1: 2
	Grade 2: 32
	Grade 3: 20
	Grade 4: 6

Abbreviations: BMI, body mass index; m, months; SD, standard deviation; y, years.

### Imaging analysis of meniscal extrusion

All patients underwent an US examination of the symptomatic OA knee for the evaluation of the medial and lateral meniscus extrusion. US evaluation was performed by radiologists with experience in musculoskeletal US and was performed using a high‐frequency linear probe 5–12 MHz. All patients were first evaluated in the clinostatism (supine) position (clino‐US) with the knee fully extended; subsequently, the examination was repeated in the orthostatism (standing) position (ortho‐US). US images were acquired for the medial meniscus on the longitudinal plane by placing the probe parallel to the medial collateral ligament, where this was most visible. At this point, the medial radial displacement of the medial meniscus was evaluated as the distance between the outermost edge of the medial meniscus to a line connecting the femoral and tibial cortical bone, as indicated by Kawaguchi et al. [17]. To evaluate the lateral meniscus, the protocol standardized by Winkler et al. [33] was used. The first step was to identify the head of the fibula and the attachment of the collateral ligament lateral to the fibula in the longitudinal plane. The probe was moved proximally following the ligament until finding its attachment on the lateral condyle femoral. At this point, the probe was translated anteriorly up to visualize the origin of the femoral insertion of the popliteus tendon in the visual field and then moved caudally to centre the probe in the joint.

The evaluation of the medial and lateral menisci extrusion was also performed by analysing a high‐resolution (1.5 T) MR imaging performed a maximum of 1 month before the US evaluation. The coronal fat sat turbo spin echo coronal proton density‐weighted sequence was used. Meniscus extrusion was measured on the central coronal slice, where the medial tibial spine was most represented. The landmark for extrusion was the osteochondral junction of the tibial plateau at the margin of the joint, paying attention to the possible presence of osteophytes. For the measurement, a reference line was plotted between the medial and lateral osteochondral junctions, defined as tibial width. Subsequently, a 90° line was drawn at the osteochondral junctions on both the internal and external sides of the knee. From here, the extrusion of the meniscus was measured parallel to the tibial width, respectively medial and lateral [20].

**Figure 1:**
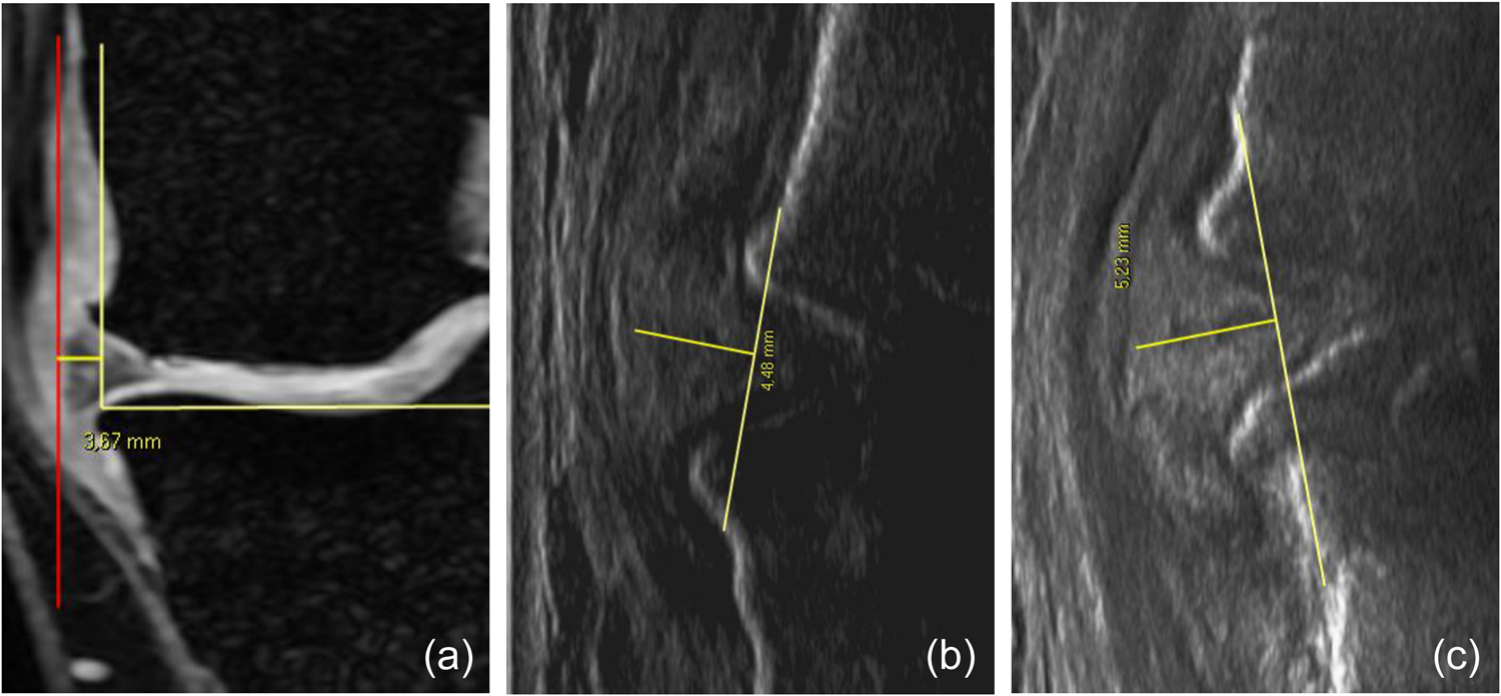
Measurement of meniscal extrusion in a male patient (52 years) using magnetic resonance (a), ultrasound in clinostatism position (b) and ultrasound in orthostatism position (c).

For the three imaging evaluations (MR, clino‐US and ortho‐US), both semi‐quantitative and quantitative measurements were performed by two experienced musculoskeletal radiologists in consensus. In particular, for the quantitative evaluation, the direct measurement of meniscal extrusion was reported in millimetres (Figure 1), while for the semi‐quantitative analysis, the classification proposed by Nogueira‐Barbosa et al. [20] was used: Grade 0 (<2 mm), Grade 1 (≥2 and <4 mm) and Grade 2 (≥4 mm). The findings of the three evaluation methods were correlated with the patient's characteristics to explore influencing factors, including age, gender, body mass index (BMI) and Kellgren–Lawrence grade.

### Statistical analysis

All continuous data were expressed in terms of the mean and the standard deviation of the mean, the categorical data were expressed as frequency and percentages and the ordinal data were expressed as median and quartiles. The Shapiro–Wilk test was performed to test the normality of continuous variables. The Levene test was used to assess the homoscedasticity of the data. The ANOVA test was performed to assess the between‐groups differences of continuous, normally distributed and homoscedastic data, the Mann–Whitney nonparametric test was used otherwise. The ANOVA test, followed by post hoc Sidak test for pairwise comparisons, was performed to assess the among‐groups differences (primary aim) of continuous, normally distributed and homoscedastic data, the Kruskal–Wallis nonparametric test, followed by post hoc Mann–Whitney test with Bonferroni correction for multiple comparisons, was used otherwise. For the secondary aim, the Pearson Chi‐square evaluated using the exact test was performed to investigate relationships between categorical variables, while the Spearman rank correlation was used to assess correlations between numerical scores and continuous data and the Kendall Tau‐b ordinal correlation was used to assess correlations between ordinal data. For the main two comparisons, with 60 patients and having an effect size equal to 0.444 and 0.428, a post hoc power equal to 0.8 was obtained. For all tests, *p* < 0.05 was considered significant. All statistical analysis was performed using SPSS v.19.0 (IBM Corp.).

## RESULTS

### Meniscal extrusion

**Figure 2:**
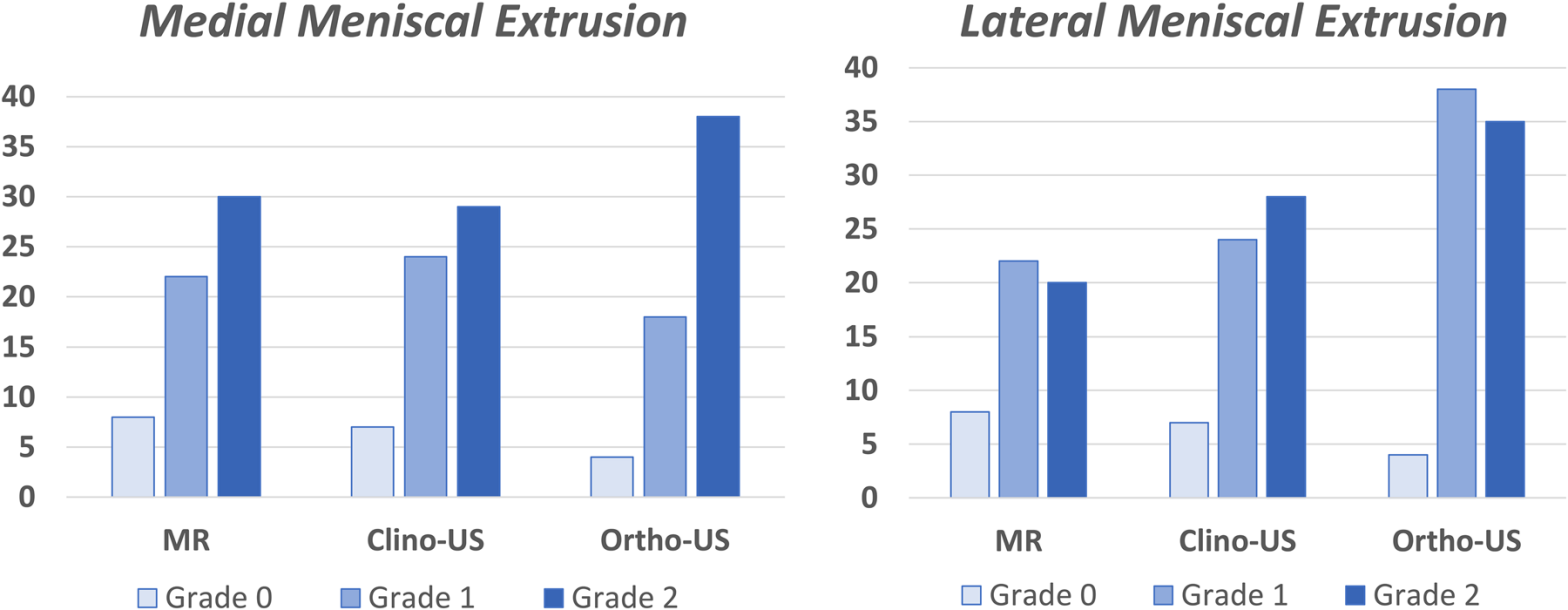
Semi‐quantitative grading of medial and lateral meniscal extrusion based on the three different imaging evaluations: magnetic resonance (MR), clinostatic ultrasound (clino‐US) and orthostatic ultrasound (ortho‐US).

The quantitative analysis documented a statistically significant higher value of the medial meniscal extrusion at the MR evaluation (4.2 ± 2.2 mm) compared to the clino‐US evaluation (4.5 ± 2.3 mm) and the ortho‐US evaluation (5.2 ± 2.3 mm) (both *p* < 0.0005). No statistically significant difference was observed between clino‐US evaluation and MR evaluation (n.s.). The semi‐quantitative analysis of the medial meniscal extrusion confirmed the same trend as reported in detail in Table 2 and Figure 2.

**Table 2:** Semi‐quantitative grading of the meniscal extrusion.

	MR	Clino‐US	Ortho‐US	MR vs. Clino‐US	MR vs. Ortho‐US	Ortho‐US vs. Clino‐US
				*p*	*p*	*p*
Medial meniscus extrusion	Grade 0: 8	Grade 0: 7	Grade 0: 4	n.s.	0.015	0.003
	Grade 1: 22	Grade 1: 24	Grade 1: 18			
	Grade 2: 30	Grade 2: 29	Grade 2: 38			
Lateral meniscus extrusion	Grade 0: 8	Grade 0: 4	Grade 0: 3	0.042	<0.0005	0.033
	Grade 1: 32	Grade 1: 28	Grade 1: 22			
	Grade 2: 20	Grade 2: 28	Grade 2: 35			

Abbreviations: Clino‐US, clinostatic ultrasonography; MR, magnetic resonance; Ortho‐US, orthostatic ultrasonography.

A statistically significant higher value of the lateral meniscal extrusion was found at the ortho‐US evaluation (4.3 ± 1.8 mm) compared to MR (3.3 ± 1.6 mm) and clino‐US (3.8 ± 1.6 mm) (both *p* <0.0005). Moreover, a statistically significant higher value of the lateral meniscal extrusion was found at the clino‐US evaluation compared to the MRI evaluation (*p* = 0.033). Both semi‐quantitative analyses of the medial and lateral meniscal extrusion confirmed the same trend, as reported in detail in Table 2.

### Patients' factors influencing meniscal extrusion

The meniscal extrusion of both medial and lateral menisci was influenced by gender. In particular, female patients had a statistically significant higher medial meniscal extrusion compared to male patients at the MR evaluation (4.9 ± 2.3 vs 3.7 ± 2.1, *p* = 0.045) and at the clino‐US evaluation (5.3 ± 2.4 vs. 4.0 ± 2.1, *p* = 0.044), while no differences were found at the ortho‐US evaluation (5.8 ± 2.5 vs. 4.9 ± 2.1, n.s.). Female patients also had a statistically significant higher lateral meniscal extrusion compared to male patients at the clino‐US evaluation (4.3 ± 1.3 vs. 3.4 ± 1.7, *p* = 0.036), while no differences were found at the MR evaluation (5.8 ± 2.5 vs. 4.9 ± 2.1, n.s.) and at the ortho‐US evaluation (5.8 ± 2.5 vs. 4.9 ± 2.1, n.s.). The differences between the values of medial and lateral meniscal extrusion obtained at MR and both clino‐ and ortho‐US evaluations were not influenced by gender.

Age significantly correlated with the extrusion of both medial and lateral meniscus. In particular, a positive correlation was found between age and medial meniscal extrusion evaluated with MR (*ρ* = 0.430, *p* = 0.001), clino‐US (*ρ* = 0.561, *p* < 0.0005) and ortho‐US (*ρ* = 0.573, *p* < 0.0005). Similarly, a positive correlation was found between age and lateral meniscal extrusion evaluated with ortho‐US (*ρ* = 0.291, *p* = 0.024), while no correlations were observed with the other two imaging methods. Age positively correlated with the difference between clino‐US and MR evaluations of the medial meniscal extrusion (*ρ* = 0.280, *p* = 0.031) and with the difference between ortho‐US and MR evaluations of the medial meniscal extrusion (*ρ* = 0.263, *p* = 0.042), with older patients showing higher differences between MR and the two US methods (Figure 2).

**Figure 3:**
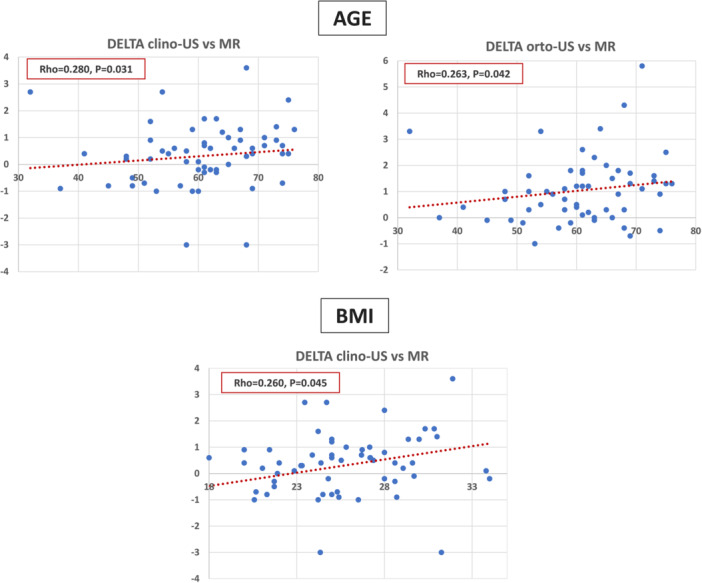
Correlation between age and body mass index (BMI) (*x*‐axis) with the differences (delta) (*y*‐axis) obtained measuring the meniscus extrusion with the different imaging methods: magnetic resonance (MR) imaging, ultrasonography in clinostatic position (clino‐US) and US in orthostatic position (ortho‐US).

BMI significantly correlated with the extrusion of both medial and lateral meniscus. In detail, a positive correlation was found between BMI and medial meniscal extrusion evaluated at clino‐US (*ρ* = 0.402, *p* = 0.001) and at ortho‐US (*ρ *= 0.366, *p* = 0.004) but not at the MR evaluation. Similarly, a positive correlation was found between BMI and lateral meniscal extrusion evaluated at clino‐US (*ρ* = 0.304, *p* = 0.018) and at ortho‐US (*ρ* = 0.316, *p* = 0.015) but not at the MR evaluation. BMI positively correlated with the difference between MR and clino‐US evaluations of the medial meniscal extrusion (*ρ* = 0.260, *p* = 0.045), with patients with higher BMI showing higher differences between MR and clino‐US evaluations (Figure 3).

## DISCUSSION

The main finding of this study is that US evaluation can identify more meniscal extrusion than the gold standard method represented by MR imaging in patients with symptomatic knee OA. Moreover, the highest values of meniscal extrusion have been documented when using US in standing position compared to the supine position, underlining the importance of the weight‐bearing assessment of meniscal extrusion in knee OA patients.

The role of meniscal extrusion is recently attracting increasing attention in the pathogenesis of knee OA. The extrusion of the meniscus may decrease its coverage area to the tibial plateau, reducing the functions normally performed by the meniscus, including shock absorption and load bearing [12]. This can lead to overstress on knee cartilage, favouring the progression of OA. In this light, meniscal extrusion was shown to be an important precursor of knee cartilage loss [3, 16], subchondral bone marrow alterations [10, 21, 32] and joint space narrowing [18, 21]. Recent longitudinal studies with large series reported that higher baseline values of meniscal extrusion in either normal knees or OA knees represent an important indicator for the development of progressive OA changes in the following years [6, 13, 21]. Both medial and lateral meniscus extrusion also showed a positive and significant correlation with clinical symptoms in patients affected by knee OA [26], and they may even influence the need for joint replacement surgery [28]. In this context, the identification and proper quantification of meniscal extrusion could be paramount in the evaluation and management of OA progression [13].

Meniscus extrusion is usually evaluated through MR imaging, which is considered the gold standard for the evaluation of meniscal disorders [4]. However, although MR imaging proved to be a useful tool in the assessment of meniscal extrusion, it is performed in supine position and, therefore, meniscus behaviour under load‐bearing cannot be evaluated [6]. Thus, US evaluation has been recently proposed for a more complete examination of the meniscal extrusion, exploiting the advantages of this approach compared to MR imaging. US assessment is not only more broadly available, timesaving, portable and cheaper compared to MR imaging, but it also allows us to perform evaluations with or without weight‐bearing [20]. US examination already showed excellent reproducibility in evaluating meniscal extrusion in a study on 11 healthy volunteers, reporting high inter‐rater and intra‐rated reliabilities [33]. In the same study, the evaluation of the meniscal extrusion with US examination demonstrated greater values (1.1 mm) compared to the MR analysis [33]. The current study confirmed these findings also in patients with knee OA evaluated with both MR and US approaches. In fact, both quantitative and semi‐quantitative analyses demonstrated significantly higher values of medial and lateral meniscal extrusion when evaluating it with US examination compared to MR imaging. Therefore, US examination can be considered a reliable technique with optimal diagnostic performance in the assessment of meniscal extrusion when compared with MR imaging. In addition, US assessment has the advantage of being performed with the joint under load, which is even more important because it allows to assess how much the meniscus extrudes during loading activities.

The importance of evaluating meniscal extrusion in weight‐bearing position has been recently recognized by the scientific community. The meniscus is a mobile structure characterized by changes in its location varying with both knee position and loading, as demonstrated by cadaveric and clinical studies [31]. It has also been demonstrated that a pathologic meniscus presents higher mobility between supine and upright positions compared to a normal meniscus [11]. Moreover, both meniscal degeneration and meniscal tears demonstrated a higher extrusion when evaluated in a weight‐bearing position [11]. Previous studies have already suggested the importance of evaluating meniscal extrusion also in weight‐bearing position. Stehling et al. [27] analysed 30 subjects (10 healthy and 20 with radiographic evidence of OA) using MR under loading or unloading conditions (applying a force on the foot), demonstrating a significantly increased meniscus extrusion under loading conditions in patients with knee OA when compared to normal subjects. Accordingly, Patel et al. [22] performed a similar study on 143 healthy volunteers and patients with knee OA, evaluating meniscal extrusion through an MR analysis with knee under unloaded and loaded conditions. These authors demonstrated a significantly higher medial meniscal extrusion when evaluating knees with MR in loaded conditions compared to unloaded conditions, while no differences in extrusion were found for the lateral meniscus. The current study confirmed the importance of evaluating meniscal extrusion in weight‐bearing conditions also using the US evaluation for knee OA patients. A statistically significant higher value of the medial and lateral menisci extrusion was found at the ortho‐US evaluation compared to both MR and clino‐US evaluations, underlining the higher diagnostic performance in identifying meniscal extrusion during loading in patients affected by knee OA.

This study also allowed us to identify factors that can influence the degree of meniscal extrusion in patients affected by knee OA. Among these, gender influenced the extent of meniscal extrusion, with women having a higher medial and lateral meniscal extrusion compared to men. This may be attributed to female physiological factors which may lead to increased laxity of knee structures such as collateral ligaments, which serve as important attachments of the menisci [34]. Moreover, age and BMI significantly correlated with the extrusion of both medial and lateral menisci, with higher values of both medial and lateral meniscal extrusions found in older patients with a higher BMI. These findings have been documented in a previous study by Achtnich et al. [1] on 75 healthy knees, which documented a meniscal extrusion depending on age and BMI. The influence of BMI on meniscal extrusion may be attributed to the increased loads transmitted to the meniscus during weight‐bearing and walking, which may promote over time a greater extrusion [30, 34]. On the other hand, ageing can lead to degeneration and deterioration of the meniscal tissue, making it less stable and resilient, thus favouring its extrusion [29]. In this regard, an interesting result obtained from this study is the positive correlation of these two demographic factors (age and BMI) with the differences observed between the meniscal extrusion measurements obtained using the two imaging approaches (MR and US). In fact, higher differences between US and MR evaluations (with higher values obtained by US) have been identified in older patients with a higher BMI. Accordingly, the evaluation of meniscal extrusion through US appears particularly important in these patients, where MR does not allow for accurate quantification and underestimates the most the extent of meniscal extrusion.

This study documented the different measurements obtained with MR and US examinations, as well as the benefits of the orthostatic evaluation, and even allowed to identify factors influencing the degree of meniscal extrusion. However, this study also presents some limitations. The sample size is limited, which represents a limitation in particular with regard to sub‐analyses. Moreover, examination remains an operator‐dependent method, although the authors relied on standardized measurement methods for measuring the meniscal extrusion. Another limitation of the study was that US measurements were performed only with the knee extended (both in weight‐bearing and non‐weight‐bearing positions). While this study set‐up was appropriate for the main study aim, the comparison with the classic MR extrusion evaluation, a dynamic US evaluation with the knee at various degrees of flexion could provide further insights to better understand meniscal extrusion. Future studies should better understand the role of meniscal extrusion in patients with knee OA also investigating the changes with both joint loading and motion, as well as the influence on symptoms and treatment response. Another interesting aspect that could be explored in future studies is the correlation between meniscal extrusion and the joint line convergence angle (JLCA), which could be useful in surgical planning of corrective osteotomy or TKA. Another limitation of this present study is the lack of multiple measurements of meniscal extrusion at different time points by different operators to assess intra‐rater and inter‐rater, respectively. Moreover, radiologists were not blinded to the outcome of the study. Finally, standardization of this type of US examination could foster a broader use in the research setting and optimize its application in the clinical practice to better evaluate meniscal extrusion in patients affected by knee OA.

## CONCLUSIONS

This study demonstrated that evaluation by US can identify more meniscal extrusion than MR imaging in patients with symptomatic knee OA. Higher extrusion values were documented in women and older patients with higher BMI, the latter being underestimated the most by the MR approach. Moreover, the highest values of meniscal extrusion have been documented using US in the standing position compared to the supine position, underlining the importance of the weight‐bearing assessment of meniscal extrusion in knee OA patients.

## AUTHOR CONTRIBUTIONS

Conceptualisation: Giuseppe Filardo, Giancarlo Facchini and Luca Andriolo. Methodology: Fabio Tortorella, Giancarlo Facchini and Maddalena Di Carlo. Data curation: Fabio Tortorella, Angelo Boffa and Luca Andriolo. Writing—original draft preparation: Fabio Tortorella and Angelo Boffa. Writing—review and editing: Giuseppe Filardo, Luca Andriolo, Giancarlo Facchini, Burt Klos and Giancarlo Facchini. Supervision: Giancarlo Facchini, Stefano Zaffagnini and Marco Miceli.

## CONFLICT OF INTEREST STATEMENT

Stefano Zaffagnini has received institutional support from Fidia Farmaceutici, Cartiheal, IGEA Clinical Biophysics, Biomet and Kensey Nash; grant support from I+ and royalties from Springer outside the submitted work. The remaining authors declare no conflicts of interest.

## ETHICS STATEMENT

This study was performed in line with the principles of the Declaration of Helsinki and approved by the Local Ethics Committee. Informed consent was obtained from each patient for study participation.

## Data Availability

The data are not publicly available due to ethical restrictions.
